# Gene print-based cell subtypes annotation of human disease across heterogeneous datasets with gPRINT

**DOI:** 10.1093/procel/pwaf001

**Published:** 2025-03-14

**Authors:** Ruojin Yan, Chunmei Fan, Shen Gu, Tingzhang Wang, Zi Yin, Xiao Chen

**Affiliations:** Department of Orthopedic Surgery of Sir Run Run Shaw Hospital, and Liangzhu Laboratory, Zhejiang University School of Medicine, Hangzhou 310011, China; Key Laboratory of Novel Targets and Drug Study for Neural Repair of Zhejiang Province, Department of Clinical Medicine, School of Medicine, Hangzhou City University, Hangzhou 310015, China; Department of Sports Medicine & Orthopedic Surgery, The Second Affiliated Hospital, and Dr. Li Dak Sum & Yip Yio Chin Center for Stem Cells and Regenerative Medicine, Zhejiang University School of Medicine, Hangzhou 310011, China; School of Biomedical Sciences, Faculty of Medicine, the Chinese University of Hong Kong, Hong Kong SAR, China; Key Laboratory for Regenerative Medicine, Ministry of Education, School of Biomedical Sciences, Faculty of Medicine, the Chinese University of Hong Kong, Hong Kong SAR, China; Kunming Institute of Zoology Chinese Academy of Sciences, the Chinese University of Hong Kong Joint Laboratory of Bioresources and Molecular Research of Common Diseases, Hong Kong SAR, China; Key Laboratory of Microbial Technology and Bioinformatics of Zhejiang Province, Hangzhou 310000, China; Department of Orthopedic Surgery of Sir Run Run Shaw Hospital, and Liangzhu Laboratory, Zhejiang University School of Medicine, Hangzhou 310011, China; Institute of Cell Biology, Zhejiang University, Hangzhou 310058, China; Department of Sports Medicine & Orthopedic Surgery, The Second Affiliated Hospital, and Dr. Li Dak Sum & Yip Yio Chin Center for Stem Cells and Regenerative Medicine, Zhejiang University School of Medicine, Hangzhou 310011, China; State Key Laboratory of Transvascular Implantation Devices, Hangzhou 310009, China

**Keywords:** disease-specific cell subtypes (DSCSs), gPRINT, cell subtypes annotation, single-cell transcriptomics, fibrosis, tendinopathy

## Abstract

Identification of disease-specific cell subtypes (DSCSs) has profound implications for understanding disease mechanisms, preoperative diagnosis, and precision therapy. However, achieving unified annotation of DSCSs in heterogeneous single-cell datasets remains a challenge. In this study, we developed the gPRINT algorithm (generalized approach for cell subtype identification with single cell’s voicePRINT). Inspired by the principles of speech recognition in noisy environments, gPRINT transforms gene position and gene expression information into voiceprints based on ordered and clustered gene expression phenomena, obtaining unique “gene print” patterns for each cell. Then, we integrated neural networks to mitigate the impact of background noise on cell identity label mapping. We demonstrated the reproducibility of gPRINT across different donors, single-cell sequencing platforms, and disease subtypes, and its utility for automatic cell subtype annotation across datasets. Moreover, gPRINT achieved higher annotation accuracy of 98.37% when externally validated based on the same tissue, surpassing other algorithms. Furthermore, this approach has been applied to fibrosis-associated diseases in multiple tissues throughout the body, as well as to the annotation of fibroblast subtypes in a single tissue, tendon, where fibrosis is prevalent. We successfully achieved automatic prediction of tendinopathy-specific cell subtypes, key targets, and related drugs. In summary, gPRINT provides an automated and unified approach for identifying DSCSs across datasets, facilitating the elucidation of specific cell subtypes under different disease states and providing a powerful tool for exploring therapeutic targets in diseases.

## Introduction

A significant challenge in the development and evaluation of clinical disease biomarkers, therapeutic targets, or reparative treatments is the lack of comprehensive information concerning the cellular composition of pathological tissues (Baldwin et al., 2023). The elucidation of the cellular basis of tissue diseases will address the knowledge gap that currently hinders the identification and implementation of efficacious therapeutic modalities in this field. In recent years, the advent of single-cell RNA sequencing (scRNA-seq) technology has facilitated in-depth exploration of gene expression patterns and functional states at the level of individual cells. Through the classification and annotation of cells, we can discern distinct cell types, thereby leveraging a more nuanced understanding of tissue pathology. Nevertheless, low-resolution cell type categorization may not be sufficient to meet the needs of the research community. To gain a more comprehensive understanding of the heterogeneity of cell populations, it is necessary to refine the categorization of cells to the level of high resolution. This will enable the identification of different cell subtypes and their gene expression characteristics in healthy and diseased states, as well as the characterization of disease-specific cell subtypes (DSCSs). This, in turn, will provide a strong foundation for the fine resolution of disease pathological mechanisms, the discovery of potential therapeutic targets, and the provision of potential therapeutic drugs.

Previous studies have identified disease-specific cellular subpopulations and molecules that play a key role in the type 2 immunopathogenesis of eosinophilic chronic rhinosinusitis with nasal polyps by single-cell RNA sequencing of individuals with different chronic rhinosinusitis (CRS) subtypes. This approach has revealed the heterogeneity of the inflammatory mechanisms of CRS (Wang et al., 2022). This study advances our understanding of immune heterogeneity and the pathogenesis of CRS subtypes, and identifies potential therapeutic approaches for the treatment of CRS and other type 2 immune-mediated diseases. Similarly, Buechler et al. found that the interstitial cell subtype structure of fibroblasts is associated with diseases such as colitis, non-small-cell lung cancer, interstitial lung disease, and fibrotic indications (Buechler et al., 2021). This could help to provide a research basis for therapeutic approaches. Secondly, the study of DSCSs can provide precise targets for the repurposing of existing drugs, the development of new drugs, and clinical trials (Huang et al., 2019). Therefore, the search for DSCSs provides an important foundation for a more comprehensive understanding of the pathogenesis and progression of diseases, which, in turn, can lead to more precise treatments.

Accurate definition of cell subtypes is essential for the reliable discovery and identification of DSCSs. Currently, the definition and classification of cell subtypes are still mainly manual. The conventional strategy is to use machine learning methods to perform unsupervised clustering of the expression profiles of cells. This is followed by the artificial assignment of the identity of each cell subcluster based on the different expression patterns of genes characterizing different cell subtypes on each cluster in the literature, as well as the functions enriched by them. In conjunction with empirical judgments (Kim et al., 2019; Svensson et al., 2017), the highly expressed genes in each cluster are considered. Given the subjectivity between the definitions of cellular subclusters in different studies, manual annotation of DSCSs leads to increased inconsistency and decreased comparability of results, which in turn limits the progress of DSCSs research. Thus, the development of automated, accurate annotation tools for DSCSs is urgently needed.

Numerous studies have aimed to develop automated cell annotation algorithms. Existing algorithms are capable of achieving cell type annotation across datasets, as well as cell subtype annotation within datasets. To illustrate, algorithms, such as scDeepsort (Shao et al., 2021) and SingleR (Aran et al., 2019) leverage information from large-scale databases (e.g., HCL [Han et al., 2020], MAC [Han et al., 2018], etc.), to annotate unknown cells at the level of major cell types. Algorithms, such as scType (Ianevski et al., 2022), CellAssign (Zhang et al., 2019a), and SCSA (Cao et al., 2020) utilize existing cell marker gene databases to annotate cell types. Algorithms, such as scMap (Kiselev et al., 2018) and Cellid (Cortal et al., 2021) facilitate cell type mapping between small-scale datasets. Additionally, the devcellpy algorithm (Galdos et al., 2022) enables cell subtype annotation on cardiac development models. However, none of the aforementioned algorithms can accomplish precise cross-dataset cell subtype annotation validation. This may be attributed to factors such as batch effects between datasets and the absence of cell subtype marker genes. Consequently, challenges persist in rapidly achieving unified labeling of disease-specific cell subtypes across datasets, necessitating the development of more suitable annotation algorithms.

To address these challenges, we propose a generalized approach for cell subtype identification with single cell’s voicePRINT (gPRINT) based on R and Python packages to accomplish a unified mapping of DSCSs obtained from any tissue that may have different annotation levels of DSCSs. The gPRINT is based on the principle of voice recognition in noisy environments (Bai et al., 2021), which first reorders the genes of each cell according to their positions in the standard human reference genome build 38 (HG38), and then maps the gene expression levels to obtain a “gene print” for each cell. Subsequently, machine learning methods were employed in accordance with the filter principle to mitigate the impact of background noise on the identification of DSCSs in single-cell analysis (Hinton et al., 2006), thereby facilitating the rapid and uniform annotation of DSCSs.

**Figure 1.**
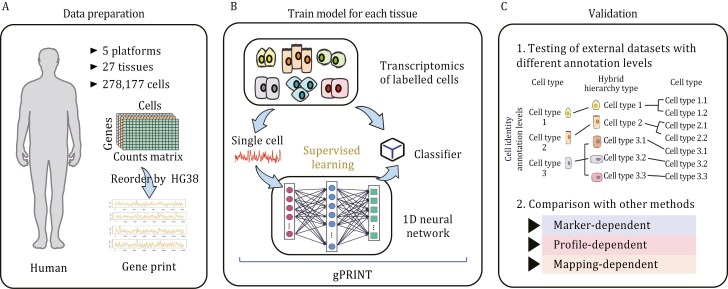
**General conceptual framework and validation of gPRINT.** (A) Selected human single-cell transcriptome profiles from HCL and other public datasets were utilized to train and validate gPRINT. The human single-cell data encompasses 278,177 cells from 27 tissues and 5 platforms. (B) For each cell, a neural network of that cell and its “gene print” was constructed for supervised learning of gPRINT using known cell labels from each tissue’s transcriptome atlas. (C) The performance of gPRINT was tested using both an internal human dataset and an external test dataset, which contains single-cell transcriptome data from multiple tissues. For the external human test dataset, gPRINT was validated at the levels of cell type, hybrid hierarchy type, and cell subtype.

To demonstrate the feasibility and robustness of gPRINT for cellular annotation, we first performed internal and external test set validation using multiple publicly available single-cell RNA sequencing (scRNA-seq) datasets and human cell landscape (HCL) database (Han et al., 2020) at the low-resolution cell type level. The datasets encompass 27 tissues, including human pancreatic tissue (Abdelaal et al., 2019; Baron et al., 2016; Muraro et al., 2016; Segerstolpe et al., 2016; Wang et al., 2016; Xin et al., 2016), colorectal tumors (Li et al., 2017), brain tissue (Camp et al., 2015), bladder, bone marrow, cervix, esophagus, heart, and ileum (Han et al., 2020), for a total of 278,177 cells (Fig. 1A). We subsequently demonstrated the capacity of gPRINT to achieve accurate annotation on datasets with higher resolution and hybrid hierarchy cell type and subtype labels (Fig. 1B and 1C). To illustrate this, we refer to the human skeletal muscle atlas (Xi et al., 2020), which contains cell labels at varying levels. Subsequently, gPRINT performed cell subtype identity annotation across diseases in a single-cell atlas of the human whole-body multi-tissue disease fibrosis atlas (Buechler et al., 2021), achieving an average accuracy of 84.23%. This accuracy surpasses that of Cellid (31.11%) and scMap (19.18%). Additionally, gPRINT provides uniform annotation of DSCSs in NSCLC diseases across datasets within the human whole-body multi-tissue disease fibrosis atlas (Buechler et al., 2021), based on the experimentally validated NSCLC atlas (Hanley et al., 2023), which includes multiple disease subtypes. This demonstrates that our algorithm effectively accomplishes high-resolution cell subtype annotation and DSCSs identification across heterogeneous datasets.

Finally, we made an attempt at auto-annotation of fibroblast subtypes on a single tissue where fibrosis likes to occur, the tendon. At the level of fibroblast subpopulations, we elucidated the subpopulation differences between pathological and normal tendons and identified cellular subpopulations specific to pathological tendons. Based on these findings, we combined with artificial intelligence deep learning models to predict potential drugs that can be used clinically to treat tendinopathies. These results demonstrate the feasibility and accuracy of the gPRINT algorithm, which can accurately and uniformly annotate DSCSs in the dataset. This provides an important foundation for in-depth investigation of the disease mechanism and screening and identification of key therapeutic targets.

## Results

### gPRINT: A tool for unified annotation of disease-specific cell subtypes based on gene prints

To address the challenge of accurately annotating cell identities within complex annotation hierarchies and achieve unified identification of disease-specific subtypes, we developed gPRINT. This is a software package based on R and Python that autonomously predicts cell features obtained from any tissue or sequencing platform. The inspiration behind the gPRINT (generalized approach for cell subtype identification with single cell’s voicePRINT) algorithm stems from the application of deep learning in sound recognition. Just as each individual possesses a unique voiceprint (Kersta et al., 1962), each cell harbors its own distinct one-dimensional gene expression pattern, termed as the “gene print”: a feature comprising both gene expression patterns and positional information. It is well established that DNA is organized in an orderly and clustered manner on each chromosome, where genes on the same nucleosome may be co-regulated, exhibiting similar expression patterns, thereby manifesting the phenomenon of “clustered expression” (Fig. S2) (Serra-Cardona et al., 2018; Wada et al., 2012). Therefore, the inclusion of gene positional information is crucial for the precise alignment of intercellular expression patterns. Furthermore, based on this, we can better predict and supplement missing values, particularly in the processing of single-cell data where dropout events are prevalent, thereby mitigating the impact of missing information. In summary, compared to traditional annotation methods that solely consider expression levels, the gPRINT algorithm, based on gene expression position and level information, theoretically enhances the accuracy of cell annotation more effectively.

The gPRINT algorithm first reorders the genes expressed in each cell according to the human reference genome sequence HG38 (see the “Methods” section). This is followed by the plotting of a unique “gene print” of each cell, based on the expression of each gene (Fig. S2). Referencing filter applications in eliminating noise and filtering out unnecessary frequencies (Chen et al., 2006), the gPRINT algorithm introduces a filtering principle in sound recognition. It treats the positional information of gene open expression as the temporal information of sound waves, considering each gene interval as a frame segment of the sound wave. Furthermore, it learns from and annotates scRNA-seq data that has been converted into waveform data, enabling improved background noise removal and enhanced accuracy through the use of a two-layer convolutional neural network (Fig. S1). In conclusion, the gPRINT algorithm is distinguished from other methods by its incorporation of not only gene expression levels but also gene positional features in its comparative analysis of cellular expression patterns. By assessing the similarity between the “gene print” of each cell in the dataset and the “gene print” of various cell subtypes in the reference dataset, it enables scalable single-cell identity mapping at the cell subtype level (Fig. S2; see the “Methods” section). Consequently, the gPRINT algorithm permits the creation of trained models based on reference scRNA-seq datasets obtained from any human tissue or sequencing platform. Comprehensive usage instructions can be found in the Methods section.

### Internal test sets for identifying cell type recognition

To optimize the model parameters, we conducted internal testing using single-cell data from 20 tissues, including Ascending Colon, Bladder, Bone Marrow, and Cervix, from the human cell landscape (HCL) database. The optimal model parameters were determined through this process: (i) ordered gene arrangement, and (ii) a CNN model (Zhang et al., 2015) with two convolutional layers. Based on these parameters, we developed the gPRINT model (see the “Methods” section).

**Figure 2.**
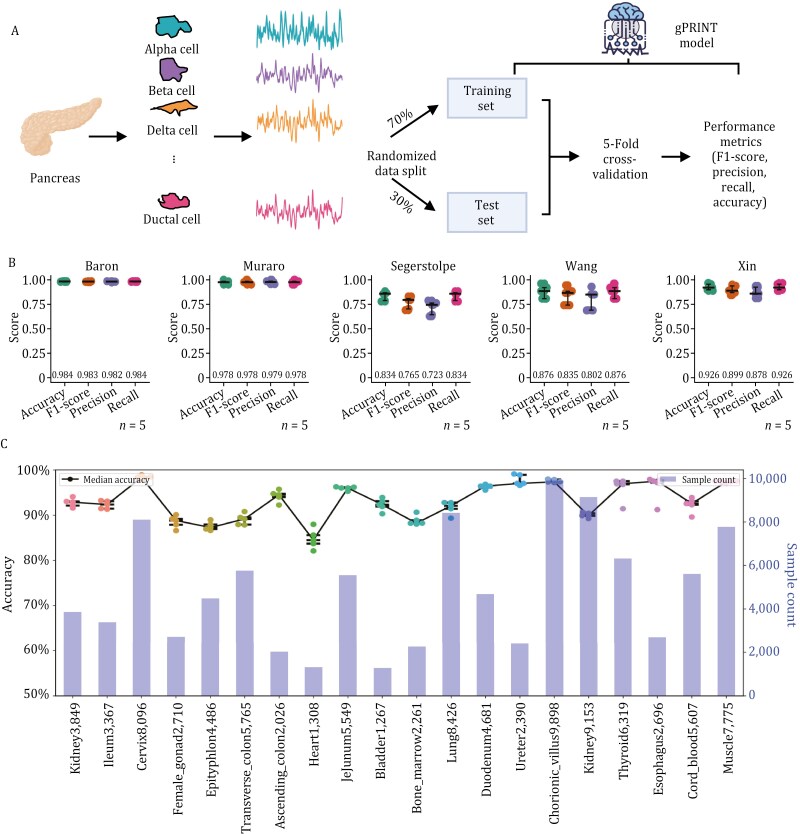
**Internal validation results of gPRINT on the public pancreas dataset and HCL database to determine the classification performance of the gPRINT algorithm.** (A) Demonstration of the gPRINT annotation scheme within pancreatic tissue. All cellular “gene prints” were randomly split into 70% as a training set and 30% as a test set, while 5-fold cross-validation was used for final testing of model accuracy. The specific evaluation metrics include F1-score, precision, recall, and accuracy. (B) Performance measures of 5-fold cross-validation for each dataset annotation are presented. Error lines indicate 95% confidence intervals. Tests were independently collapsed for *n* = 5. (C) Classification accuracy of gPRINT’s internal annotations on the HCL database is shown. The left side of the axis is the correct rate, and the right side represents the number of cells.

**Figure 3.**
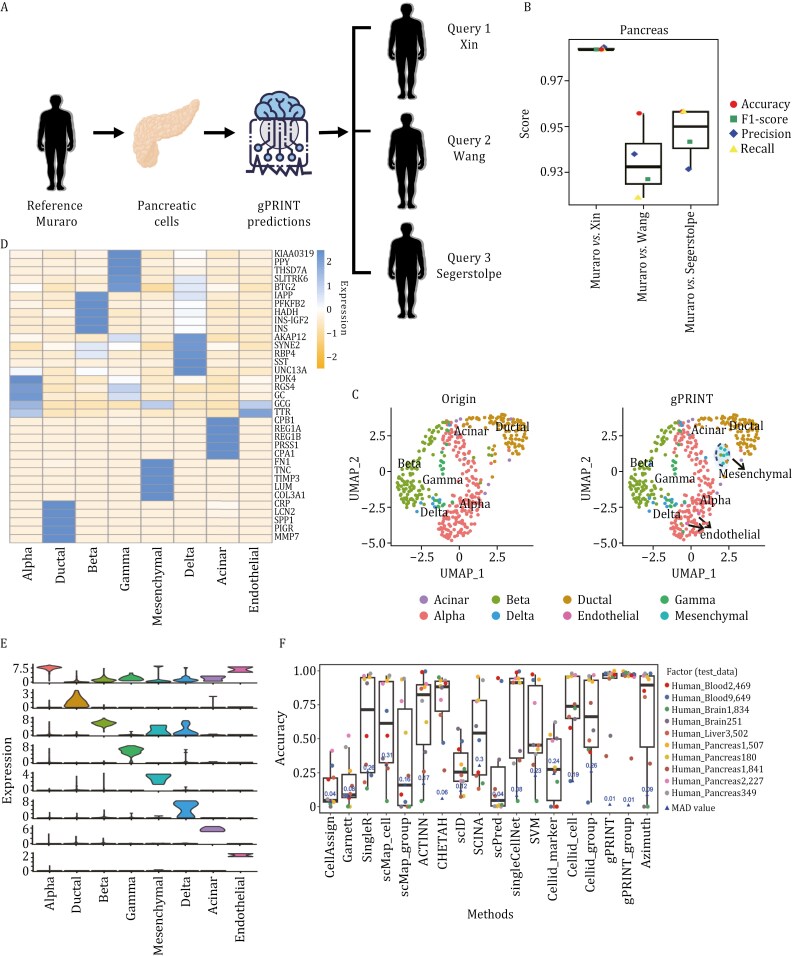
**Illustration of the performance of gPRINT cell type identity mapping from different single-cell RNA sequencing (scRNA-seq) datasets within the pancreas and other tissues.** (A) Demonstration of the cross-tissue annotation scheme of gPRINT within pancreatic tissue. The Muraro dataset was used as a reference to complete the annotation of the Xin, Wang, and Segerstolpe datasets. (B) Boxplot of assessment indicators for annotated results for different datasets. Boxplots summarize the method’s F1-scores, which indicate the proportion of correctly classified cells. The median (center line), interquartile range (hinges), and 1.5 times the interquartile range (whiskers) are displayed. (C) UMAP plots of cells from the Wang dataset are shown. On the left are the cell types defined in the original article, and on the right are the results of cell type prediction based on gPRINT, where the colors of the cells represent different cell types. (D) Mean values of the top five differentially expressed markers in all gPRINT-predicted cell types were obtained and presented as heatmaps. (E) Violin plots representing the expression levels of marker genes for all different cell types were generated. (F) A histogram of metrics evaluated for each annotation algorithm on the multiple tissue datasets was constructed. Boxplots summarize the method’s F1-scores, displaying the median (center line), interquartile range (hinges), and 1.5 times the interquartile range (whiskers). Additionally, the Median Absolute Deviation (MAD) values are included to indicate the variability and robustness of classification performance across different datasets.

Then, to assess the feasibility of gPRINT, we initially utilized a set of gold standard datasets from human pancreas samples (Wang [2016], Xin [2016], Muraro [2016], Baron [2016], and Segerstolpe [2016]) to conduct a preliminary test of low-resolution cell-type annotation within the dataset. Specifically, we first converted the cellular gene expression matrices of each dataset into a “gene print” expression format, and then randomly partitioned the data into a 70% training partition and a 30% test partition. On the 30% partition, we performed a five-fold cross-validation. Four key performance metrics were evaluated, including model accuracy, precision, recall, and F1-scores (Fig. 2A, see the “Methods” section). For cell annotation in the Baron dataset, the model demonstrated high overall accuracy, reaching ~98.4% over five independent training rounds (Fig. 3). The model also exhibited high sensitivity, with a recall value of 98.4% ± 0.3% and an overall precision of 98.2% ± 0.4%. Similarly, gPRINT performs impressively on other pancreas datasets, with an average accuracy of 97.8% on the Muraro dataset, 83.4% on the Segerstolpe dataset, 87.6% on the Wang dataset, and 92.6% on the Xin dataset. The average accuracy was 92.6% (Fig. 2B). These results collectively demonstrate the viability of the gPRINT algorithm for high-precision cell identity prediction. For instance, a 5-fold cross-validation on 30% of the test set on the Muraro dataset confirmed the high accuracy of the predictions. The prediction accuracy exceeded 93% for all cell categories, with four categories, acinar cells and alpha cells, exceeding 99%. The confusion matrices for the remaining datasets are presented in Fig. S3A. We conducted another internal validation on single-cell datasets from human colorectal tumors (Li dataset [Li et al., 2017]) and human brain tissues (Camp dataset [Camp et al., 2015]), resulting in accuracy rates exceeding 92% (Fig. S3B). Furtherly, we also performed internal validations on one of the HCL database, including the bladder, bone marrow, cervix, esophagus, heart, and ileum (Fig. 2C), with accuracy rates exceeding 80%. These validations collectively demonstrate the feasibility of gPRINT across various tissues and datasets. These results demonstrate the feasibility of gPRINT in cell type annotation.

### Aligning cell type identities across datasets within the same tissue

To further evaluate the robustness of gPRINT, we compared the annotation effectiveness of gPRINT for scRNA-seq datasets sourced from different sequencing platforms (Fig. 3A, Table 1). We employed the gold standard dataset of human pancreas samples for cell type annotation across datasets. This dataset encompasses several commonly used single-cell transcriptome sequencing platforms, including the CEL-Seq2 (Muraro dataset), SMARTer (Wang and Xin datasets), and Smart-Seq2 (Segerstolpe dataset). The gPRINT annotation across datasets is obtained by learning the “gene print” from the reference dataset as a reference to match against other test datasets. In this study, the Muraro dataset with a larger number of cells and clearer cell type labels was employed as a reference to annotate other pancreas datasets (Table S1). The gPRINT consistently yielded high precision (> 93%), accuracy (> 95%), recall (> 91%), and F1 values (> 92%) across all evaluated reference-to-query datasets. Furthermore, across all evaluated reference-to-query cell-type assignments (Fig. 3B), the gPRINT demonstrated high performance. In conclusion, gPRINT is capable of accurately mapping single-cell labels at the cell-type level for low-resolution across datasets.

**Table 1.** Here provides specific information on the datasets pertaining to pancreatic tissues.

Tissue	Cell number	Gene number	Accession number	Protocol	Name
Pancreas	2,126	19,140	GSE85241	CEL-Seq2	Muraro
Pancreas	3,514	25,525	E-MTAB-5061	Smart-Seq2	Segerstolpe
Pancreas	635	19,950	GSE83139	SMARTer	Wang
Pancreas	1,600	39,851	GSE81608	SMARTer	Xin

Specifically, the Muraro data obtained by sequencing with the CEL-Seq2 method was used as a reference, and the Wang dataset obtained by sequencing with the SMARTer method was employed as an example of annotation. The gPRINT was utilized to successfully obtain the annotation results of cell types, including acinar, beta, gamma, alpha, delta, ductal, and others. Additionally, the software was employed to annotate mesenchymal and endothelial populations that were not previously defined in articles (Fig. 3C). Furthermore, the annotations assigned during the atlas construction were validated by performing differential gene expression analysis for all major annotated cell types. This analysis confirmed the unique expression of major cellular markers reported in the eight major cell populations identified (Fig. 3D). Subsequently, violin mapping was conducted with marker genes for cell types that were re-annotated using gPRINT (Fig. 3E). This analysis revealed that the re-annotated cell populations exhibited high expression of marker genes for mesenchymal and endothelial cells, which validated the gPRINT annotation and demonstrated that gPRINT corrected for the cell types in the original dataset, thus completing the cross-dataset annotation.

Subsequently, gPRINT was applied to single-cell data derived from HCL’s blood, brain, liver, pancreas, and other tissues. The mean and median accuracy values of the gPRINT algorithm (91.15% and 97.16%, respectively) were found to be higher than those of other algorithms, including Azimuth (67.95% and 89.51%, respectively). The standard deviation of gPRINT was 0.1959, which was lower than that of other algorithms with mean accuracies greater than 20%. These findings demonstrate that, in comparison to other algorithms, including marker gene-based cell annotation algorithms (e.g., CellAssign [Zhang et al., 2019a], Garnett [Pliner et al., 2019], and SCINA [Zhang et al., 2019b]) and reference dataset-based cell annotation algorithms (e.g., scMap [Kiselev et al., 2018], SingleR [Aran et al., 2019], ACTINN [Ma et al., 2020], CHETAH de Kanter et al., 2019], scID [Boufea et al., 2020], scPred [Alquicira-Hernandez et al., 2019], SVM [Schuldt et al., 2004], Cellid [Cortal et al., 2021] and Azimuth), the gPRINT algorithm has been demonstrated to have high accuracy, minimal fluctuation, and stable algorithm performance (Figs. 3F and S4; Table S2). The above results demonstrate that the gPRINT algorithm can be utilized to compare datasets of similar biological origin collected by different laboratories. It ensures consistency in annotation and analysis, and eliminates the influence of batch effects on the analysis.

### Mapping the human skeletal muscle atlas based on complex cell type hierarchies

The efficacy of the gPRINT algorithm for identity mapping at low-resolution cell type levels was evident. Subsequently, the gPRINT algorithm was employed to further assess its annotation accuracy on cell identity labels with a hybrid hierarchy type of different levels. This allowed for the speculation of the feasibility of gPRINT in annotating high-resolution cell subtypes.

**Figure 4.**
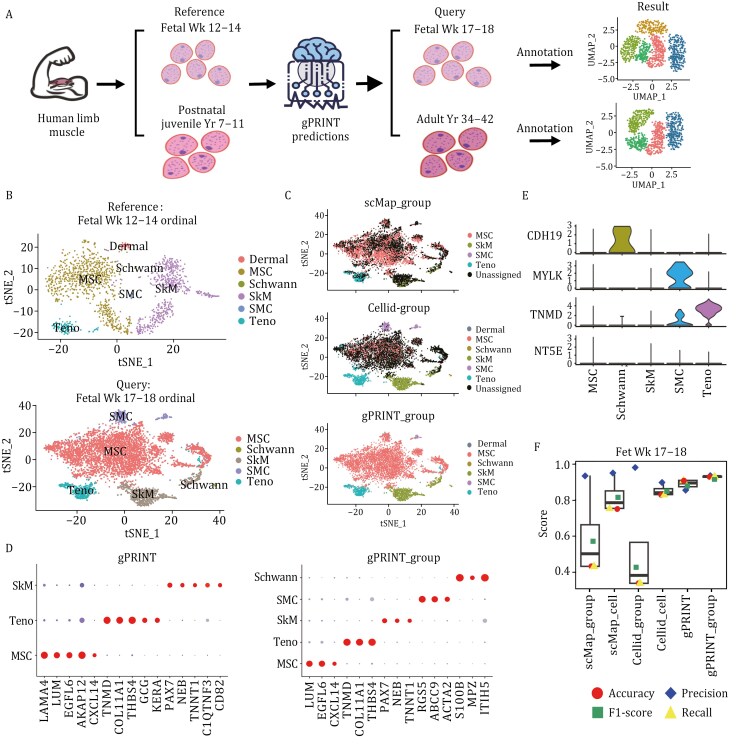
**Performance of gPRINT on human fetal limb skeletal muscle atlases in terms of cell type identity mapping.** (A) Demonstration of gPRINT’s cross-sample annotation scheme across time on human limb skeletal muscle atlases. Fetal Wk 12–14 samples were used as reference data and fetal Wk 17–18 samples were used as test data on the fetal skeletal muscle atlas; postnatal juvenile Yr 7–11 samples were used as reference data and adult Yr 34–42 samples were used as test data on the adult skeletal muscle atlas. (B) t-SNE plots of cell distribution of different samples on the fetal limb skeletal muscle atlas, with cell color representing cell type. (C) t-SNE plot demonstrating the annotation results of different annotation methods on fetal Wk 17–18 samples, with color of cells representing cell type. (D) Mean values of the top four or three differentially expressed markers in all gPRINT and gPRINT_group algorithms predicted to be obtained by the gPRINT and gPRINT_group algorithms in the cell types and presented as a dot plot. (E) Violin plots representing the expression levels of marker genes for all different cell types. (F) Boxplot of evaluation metrics for each annotation algorithm on fetal Wk 17–18 samples. Boxplots summarize the method’s F1–scores, displaying the median (center line), interquartile range (hinges), and 1.5 times the interquartile range (whiskers).

First, the Fet Wk12–14 and Fet Wk17–18 datasets (Xi et al., 2020), which pertain to the human fetal limb skeletal muscle atlas in the human skeletal muscle atlas dataset GSE147457 (Xi et al., 2020), were annotated using a marker gene database-based approach (Fig. 4A). It was found that the scType and Cellid algorithms, which are based on the marker gene pool, were unable to accomplish effective annotation. This may be an annotation failure caused by the lack of marker genes in the signature gene pool that originally defined each cell subpopulation on the dataset (Fig. S5). Subsequently, the Fet Wk12–14 and Fet Wk17–18 datasets on the human fetal limb skeletal muscle atlas were annotated using the SingleR algorithm, a method based on large reference databases. The results demonstrated that SingleR also failed to accomplish effective annotation. This may be attributed to the lack of marker genes of these cell subtypes in large reference databases (Fig. S5).

In light of the aforementioned issues, including the dearth of information on feature genes and subcellular subtypes in extensive databases, we employed two additional algorithms based on projecting new samples to an existing reference dataset: scMap and Cellid, with the objective of annotating across datasets the Fet Wk12–14 dataset, which serves as a reference for Fet Wk17–18. The results demonstrate that these two algorithms outperform methods that rely on characterized genomic databases or large reference datasets. The algorithms can reproduce the cell labels defined in the original study, particularly in the case of single-cell type-based learning (Fig. 4C). The Fet Wk12–14 dataset includes cell labels for Schwann cells, tenocytes (Teno), mesenchymal stem cells (MSCs), and muscle cells, where muscle cells include smooth muscle cells (SMCs) and skeletal muscle cells (SkMs) (Fig. 4B). However, there were still numerous cells that could not be clearly defined (illustrated as black dots in the t-SNE plots), and in some instances, SMCs were erroneously identified as MSCs.

We then evaluated the performance of our gPRINT and gPRINT_group algorithms against scMap and Cellid using specific performance metrics (Fig. 4F). As evidenced by the box plots, swarm plots, and confusion matrices for each cell type, gPRINT and gPRINT_group demonstrate superior performance in terms of accuracy, recall, and F1-score when compared with the other two algorithms. In particular, gPRINT and gPRINT_group consistently demonstrate superior performance in terms of accuracy (exceeding 90% and 92%, respectively), recall (exceeding 90% and 92%, respectively), and F1-scores (exceeding 87% and 91%, respectively) across all evaluated reference-query cell-type assignments. The second-best algorithm in this case was Cellid_cell, which was evaluated on specific metrics such as accuracy (> 82%), recall (> 82%) and F1 value (> 84%). In this instance, the gPRINT_group algorithm proved to be a more accurate method than the gPRINT algorithm. Further examination of the violin plots, which illustrate the expression of genes that distinguish different cell types on gPRINT_group-annotated isoforms (Fig. 4E), revealed that *CDH19*, *MYLK*, *TNMD*, and *NT5E* are genes that are characteristic of Schwann cells, SMCs, Teno, MSCs, respectively (Xi et al., 2020). The marker genes are highly expressed on the corresponding cell types predicted by the gPRINT_group method, demonstrating that the gPRINT_group algorithm can accomplish the mapping of cell types and subtypes across time from the 12–14 to 17–18 weeks dataset on the human fetal skeletal muscle atlas with high accuracy. This suggests that the gPRINT algorithm is able to successfully solve the problem of mixing different levels of cell identity labeling.

Subsequently, the gPRINT algorithm was validated across datasets (Xi et al., 2020) on postnatal juvenile (7–11 years) and adult (34–42 years) datasets from adult limb skeletal muscle atlases (Figs. 4A and S6A). The results show that gPRINT and gPRINT_group outperform other algorithms in terms of accuracy, recall, and F1-score. Specifically, the gPRINT and gPRINT_group consistently demonstrate high accuracy (greater than 92% and greater than 93%, respectively), recall (greater than 92% and greater than 93%, respectively), and F1 values (greater than 91% and greater than 93%, respectively) across all evaluated reference-query cell type assignments. In this case, the second-best algorithm is Cellid_cell, which has been specifically evaluated for accuracy (> 64%), recall (> 64%), and F1 value (> 74%). Additionally, the annotated cell subtypes showed high expression of their respective marker genes (Fig. S6E). These findings demonstrate that gPRINT is capable of accurately mapping cell types and subtypes across time in human fetal skeletal muscle atlases containing mixed cell types and subtypes. Therefore, it presents a viable approach for high-resolution cell subtype annotation.

### Matching the systemic multi-tissue fibrosis-related disease atlas

The gPRINT algorithm has already demonstrated its potential in accurately predicting cell subtype labels. Here, we further validate the performance of the gPRINT algorithm in matching labels at high-resolution cell subtype levels. Given that differences between healthy and diseased states may obscure or interfere with true differences between cell subtypes, it can be challenging to accurately map cell subtypes between healthy and diseased subgroups. Similarly, batch effects across different datasets can also disrupt the accuracy of cell subtype mapping. Consequently, achieving unified annotation of DSCSs across heterogeneous datasets remains a significant hurdle.

Fibrosis is a major area of research interest, as it affects nearly all human organs, compromising tissue structure and function, and posing serious health risks (Aggarwal et al., 2024; Henderson et al., 2020; Mutsaers et al., 2023; Wynn et al., 2008; Younesi et al., 2024). Identifying key therapeutic targets linked to fibrosis and developing effective anti-fibrotic drugs is critical. We attempted to perform high-resolution cell subtype label matching on whole-body multi-tissue disease atlas (Buechler et al., 2021) and cell label mapping across datasets as a means of identifying key DSCSs under different diseases associated with fibrosis for more targeted drug recommendations.

**Figure 5.**
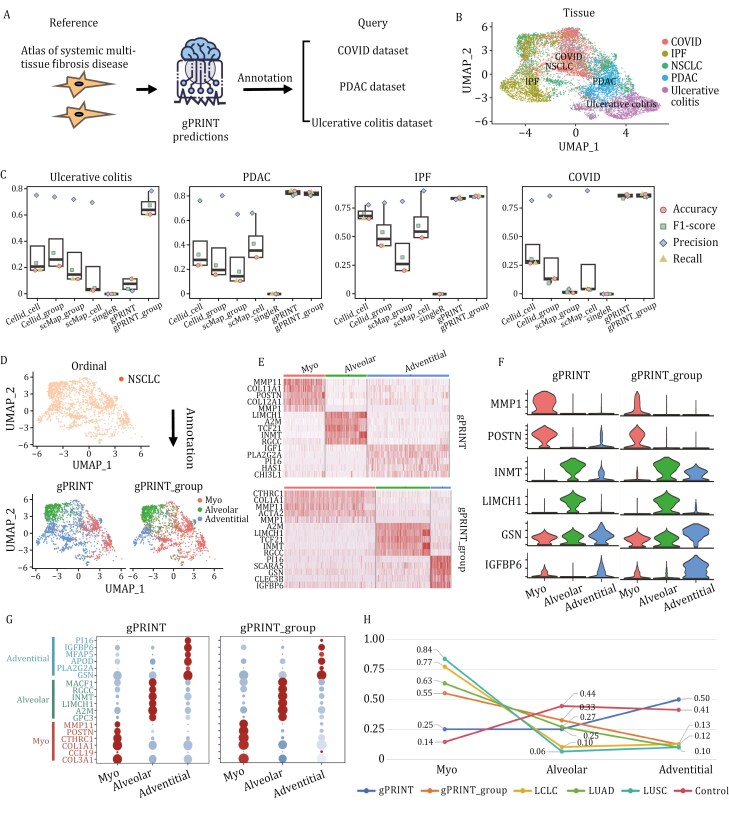
**Matching cell subtype profiles across systemic multitissue fibrosis-related diseases.** (A) gPRINT scheme of systemic multitissue cell subtypes across diseases. (B) UMAP plot of cell subtype distribution of systemic multitissue-associated fibroblasts. Cell colors represent cell subtypes. The left side represents the single-cell NSCLC fibroblast dataset from the original article, and the four on the right represent different samples by disease subgroup. (C) Box plots summarizing the F1-scores of the method and showing the median (centerline), interquartile range (hinge), and 1.5-fold interquartile range (whiskers). (D) UMAP plot showing the results of annotating fibroblasts in the Matthew database using the gPRINT and gPRINT_group annotation methods, where the color of the cells represents the cell subtype. (E) The expression of the top 5 differentially expressed markers in different cell subtypes is labeled and presented as a heatmap. (F) Dot plots showing the expression of marker genes in different cell subtypes annotated by different algorithms. (G) Violin plots showing the expression levels of marker genes for all different cell types on cell subtypes obtained by different annotation methods. (H) Line plots of the proportions of cell subtypes plotted under different disease subgroups and annotation methods. Different colors represent different datasets.

First, we compared the performance of different mapping algorithms by cross-validating across disease datasets within the human whole-body multi-tissue disease fibroblast atlas (Buechler et al., 2021). The atlas comprises cancer-associated fibroblasts derived from pancreatic ductal adenocarcinoma, colon fibroblasts from patients with non-small-cell lung cancer (NSCLC), ulcerative colitis, idiopathic pulmonary fibrosis, and lung fibroblasts from patients with COVID-19 (Fig. 5). The NSCLC group had the highest sequencing quality and balanced cell type distribution (Fig. S7) and was used as a reference for mapping cell subsets in other groups. Comparing the annotation results of the different algorithms on the fibroblast dataset for each disease state, we found that gPRINT and gPRINT_group consistently yielded high accuracy (> 81% and > 80%), recall (> 82% and > 79%) and F1 values (> 80% and > 77%, respectively), across all evaluated reference-to-query cell-type assignments. The Cellid_cell algorithm, which is specifically evaluated for the following metrics, ranks second only to the gPRINT algorithm. These metrics include accuracy (> 23%), recall (> 23%), and F1 values (> 30%) (Fig. 5). These findings suggest that the gPRINT algorithm is an effective approach for accurately mapping cell subtypes and can facilitate cell type mapping across disease states.

To further validate gPRINT, we conducted cross-validation using the NSCLC disease atlas, which was experimentally validated by multiplex immunohistochemistry and digital cytometry (CIBERSORTx). As this systematic atlas had not been subjected to experimental validation, we undertook a further mapping exercise using a human NSCLC disease atlas (Hanley et al., 2023), which had been validated by multiplex immunohistochemistry and digital cell counting method (CIBERSORTx). The atlas encompassed both healthy and disease subgroups, including control samples, lung adenocarcinomas (LUAD), squamous cell carcinomas (LUSC), and large cell carcinomas (LCLC) (Fig. S8A). The results showed that gPRINT can accurately identify DSCSs and map cell subtypes across datasets (Fig. S8). We observed that the proportion of myofibroblasts was higher in the NSCLC disease subgroup, while alveolar cells were dominant in LUAD compared with the LCLC and LUSC subgroups (Fig. 5H). This suggests that different cell subtypes are associated with different NSCLC subgroups, which is helpful for the discovery of targeted drugs.

Ultimately, the experimentally validated human NSCLC disease atlas was employed as a reference for the re-annotation of the NSCLC group within the whole-body multi-tissue fibroblast atlas. Through cross-validation between these two atlases, we were able to confirm the feasibility of gPRINT in accurately mapping cell subtypes across datasets. Specifically, three distinct fibroblast subtypes were identified (Fig. 5D), with the annotated differential gene heatmap (Fig. 5E) and the expression bubble and violin plots (Fig. 5F and 5G) confirming the accuracy of these annotations. Further analysis showed that the proportion of fibroblast subtypes in the NSCLC group was closely related to that in the LUAD subgroup, suggesting that more precise treatment strategies are needed for this disease subtype. At the same time, gPRINT also annotated cell subtypes across datasets for breast cancer, and the results demonstrated the feasibility of gPRINT annotation of cell subtypes in different diseases (Fig. S9).

In conclusion, the gPRINT algorithm demonstrated remarkable proficiency in cell subtype annotation across a diverse array of disease datasets. Its exceptional performance was particularly evident in high-resolution mapping across datasets and disease states, with its accuracy and reliability having been rigorously validated. The algorithm is capable of elucidating differences in cell subtypes composition across disease subgroups and of identifying DSCSs, thus providing a new basis for developing targeted treatment strategies. Therefore, gPRINT holds great potential for future disease research and targeted therapy exploration, offering strong support for further investigations into cell subtypes and their clinical applications.

### Annotating tissue-specific fibrosis disease atlases

The matching of cell subtype labels in the human whole-body multi-tissue disease fibrosis atlas allows us to gain a deeper understanding of the characteristics and functions of fibroblasts in the associated different fibrosis-related disease states. This provides more precise guidance and direction for disease treatment and prevention. We then turn our attention to the target organ of fibrosis, the tendon. Tendon is a class of fibroblast-based tissues, and as an important component of the locomotor system, tendinopathy has become a significant public health problem that requires urgent attention. According to statistics, tendon injuries account for approximately 30% of sports injuries (Docheva et al., 2015). Nevertheless, the current treatment for tendinopathy is still based on symptomatic treatments such as pain relief and the absence of allopathic therapies. This is mainly due to the lack of a clear pathological mechanism. The precise localization of pathological subgroups of tendinopathies is important for the development of clinical therapies for tendinopathies.

Although several cases of single-cell transcriptome sequencing in tendinopathy have been reported (Fu et al., 2023; Kendal et al., 2020), the shortcomings of existing algorithms in annotating fibroblast subpopulations have resulted in the data being largely manually annotated, with cell type definitions varying widely from study to study. The achievement of automated and accurate annotation of fibroblast subtype labels in tendinopathies would facilitate rapid and labor-saving insights into the functions and characteristics of different subtypes of fibroblasts in tendinopathies. This would assist in the discovery of new therapeutic targets and treatments.

**Figure 6.**
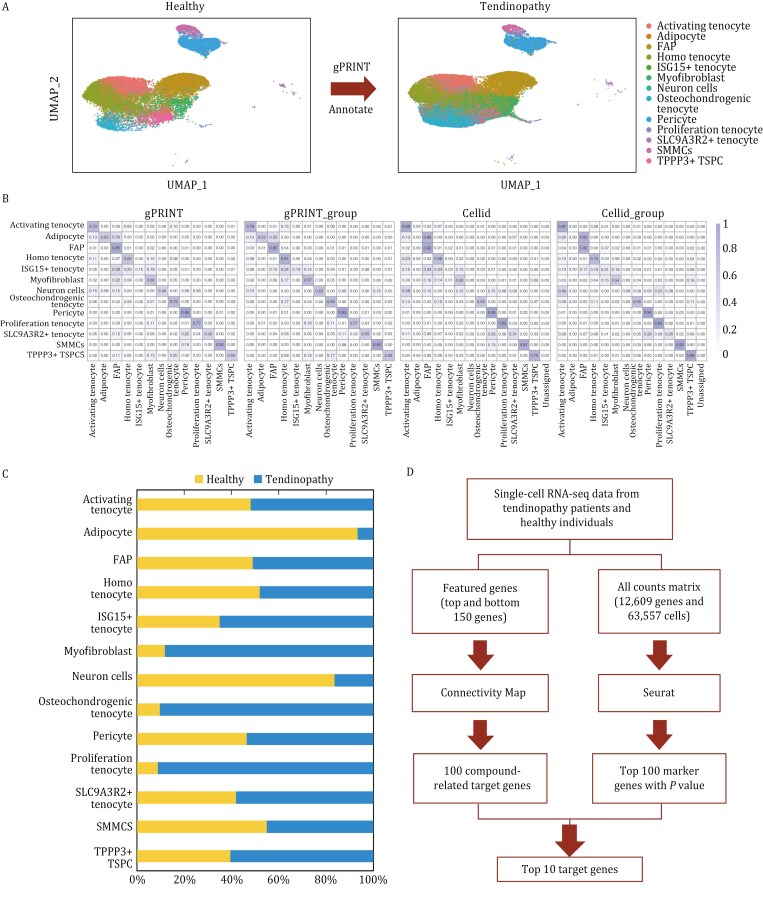
**Fine subtype annotation and prediction of key target genes under tendinopathy fibroblasts.** (A) Human cell subtype mapping across tendinopathy and healthy groups. Identity mapping of cellular subtypes on the tendinopathy dataset was accomplished using single-cell data from the healthy group as a reference. (B) Confusion matrix representations comparing manual annotations with the classification labels generated by gPRINT, gPRINT_group, Cellid and Cellid_group. The diagonal represents the percentage of labels that agree between both classification methods. (C) A bar graph showing the percentage of cell subtypes mapped by the gPRINT approach. (D) The process of identifying key target genes and conducting related drug screening based on the sought-after DSCSs.

Consequently, we implemented an automated and precise methodology for the annotation of fibroblast subtype labeling in human tendinopathies, utilizing the gPRINT algorithm. First, a single-cell atlas was constructed containing fibroblasts from patients with tendinopathies. This included data from four healthy individuals and six patients with tendinopathies, with a total of over 60,000 cells. The atlas was then manually annotated, resulting in the identification of a total of 13 fibroblast subtypes. Subsequently, we proceeded to map the characteristics of the cell subtypes on the atlas. The objective was to ascertain the identity of the patient’s fibroblasts by developing a fibroblast identity prediction model based on the subtype labels of healthy individuals using gPRINT and other cell mapping algorithms (Fig. 6A). The gPRINT and gPRINT_group algorithms demonstrated superior performance compared to the Cellid and Cellid_group algorithms, as evidenced by the confusion matrix corresponding to manual annotation (Fig. 6B). Consequently, the gPRINT algorithm was selected for the prediction of fibroblast subtypes in the newly collected single-cell data on tendon diseases, which significantly reduced the effort required for annotation. Furthermore, the established tendinopathy fibroblast atlas can be extended for uniform annotation by incorporating the newly acquired single-cell data.

Although the types of fibroblast subpopulations were essentially the same in normal and pathological tendons, the proportions of osteochondrogenic tenocyte cell subtypes, myofibroblast subtypes, and proliferative tenocyte cell subtypes were higher in pathological tendons. This led to the hypothesis that an increase in the number of cells of these three subtypes may contribute to the development of tendon disease.

To further explore potential treatments for tendon disorders, we conducted a search for compounds that inhibit the expression of osteochondrogenic tendon cell and myofibroblast subtypes using the drug database CMap. CMap is a gene expression profiling database based on the regulation of gene expression, developed by the Broad Institute. It is used to reveal functional links between small molecules, genes, and disease states. By intersecting marker genes for two key subtypes that are significantly increased in pathological tendons with target genes predicted to be relevant drugs in CMap, we can identify potential drugs that have the potential to target and regulate specific cellular processes associated with these fibroblast subtypes (Figs. 6D and S10). Specifically, we selected the top 150 up- and down-regulated genes from the single-cell RNA sequencing data of tendon disorders and healthy individuals as featured genes (Tables S3 and S4), which were then entered into Connectivity Map (CMap) for querying. The obtained normalized connectivity score values associated with the CMap algorithm were used to generate two-dimensional coordinate plots (Fig. S10; Table S5). The connectivity score is a measure of the correlation between drug perturbation features and disease perturbation features. A positive connectivity score indicates that the expression profile perturbation of the drug is positively correlated with the expression profile perturbation of the disease, suggesting that the drug may induce or exacerbate the disease state. Conversely, a negative connectivity score indicates that the expression profile perturbation of the drug is negatively correlated with the expression profile perturbation of the disease, suggesting that the drug may attenuate or even reverse the disease state. Thus, by calculating the connectivity score between a drug and a disease state, the effect of the drug on the disease state can be assessed and the therapeutic effect predicted. Conversely, a drug with a negative connectivity score indicates that the expression profile perturbation of the drug is negatively correlated with the expression profile perturbation of the disease, suggesting that the drug may attenuate or even reverse the disease state. Thus, by calculating the connectivity score between a drug and a disease state, the effect of the drug on the disease state can be assessed and the therapeutic effect can be predicted. Consequently, the drugs that we are seeking are the 100 small molecule drugs depicted in gray in Fig. S10 (Table S6). Our objective is to achieve precision therapy by targeting specific subtypes of tendon disease.

By cross-referencing the CMap-predicted target genes of relevant small molecule drugs with marker genes of the two subtypes, we obtained a list of relevant target genes for the treatment of tendon diseases. These target genes include *MAP3K2*, *CALM1*, *MAP2K2*, *FKBP1A*, *MAPK1*, and others. We also identified drugs that inhibit the expression of these genes, such as axitinib, sirolimus, and perphenazine. In conclusion, these results suggest that the gPRINT algorithm can be used to predict the cellular subtypes of tendon fibroblasts with great precision, both in healthy and diseased states. This can serve as a reference for future studies on tendon fibroblasts and lay the foundation for precise treatment of tendon diseases at the fine cell subtype level.

## Discussion

In this study, we introduced the concept of a single-cell “gene print” expression pattern. Using this pattern and a neural network algorithm, we developed the gPRINT tool for uniform annotation of DSCSs. Since genes often show clustered expression patterns (Fig. S2) and there may be correlations between neighboring genes, we sorted the gene expression matrix according to HG38. This ordering allowed us to obtain a one-dimensional “gene imprint” expression pattern unique to each cell. In contrast to the matrix format, this pattern incorporates positional information between genes, thereby more closely approximating actual gene expression. In this model, by applying principles analogous to those employed in speech recognition filtering, such as the addition of a convolutional layer, we can more effectively address the batch effect, analogous to noise reduction in speech recognition, and ultimately enhance the accuracy and reliability of cell type and subtype identification. Consequently, the gPRINT algorithm, which is based on a neural network model and a “gene print” expression pattern, effectively addresses the issues of cell heterogeneity and noise in single-cell RNA sequencing data, thereby enhancing the accuracy and reliability of cell type and subtype recognition.

Specifically, in this study, we first validated the feasibility of our own algorithm on pancreatic tissues and internal test sets of various types of tissues in the HCL database. We then proceeded to successfully complete cell type mapping across datasets on pancreatic tissues and compared it with other algorithms. In an external validation study on publicly available pancreatic datasets, the gPRINT algorithm demonstrated a high level of accuracy, with an average accuracy of 97.39%. Moreover, when externally validated on datasets from the HCL database, including pancreatic, liver, brain, and blood datasets, gPRINT demonstrated superior performance compared to other algorithms. The median accuracy of gPRINT was 97.16%, with a MAD value of 0.0098, ranking first in both cases. The second-best performer was Azimuth, with a median accuracy of 89.51% and a MAD value of 0.085. These results indicate that the gPRINT algorithm achieves higher accuracy in cell type annotation than other cell labeling algorithms, with smaller bias and greater robustness. This suggests that the gPRINT algorithm may be capable of reaching the performance levels of existing algorithms, or even exceeding them, in the context of cell type annotation.

Subsequently, we proceeded to complete the mapping of cell identity labels across datasets with varying levels of annotation. In the annotation of the human fetal skeletal muscle atlas, the gPRINT and gPRINT_group algorithms mapped the cell type and subtype annotations across the 12–14 weeks data to the 17–18 weeks data, and both of them maintained a high accuracy (> 90% and > 92%), which was followed by the gPRINT algorithm for the Cellid_cell algorithm, which was evaluated by the accuracy (> 82%). The gPRINT algorithm exhibited the highest accuracy (92%), followed by the gPRINT algorithm for the Cellid_cell algorithm, which was evaluated by the accuracy (82%). With regard to the annotation problem of adult limb skeletal muscle atlas, we further completed the mapping of cell identity labels across different annotation levels of the data set. The gPRINT and gPRINT_group algorithms mapped cell type and subtype annotations across youth data from 7–11 years of age to adult data from 34–42 years of age with high accuracy, exceeding 92% and 93%, respectively. The gPRINT algorithm exhibited comparable performance, with accuracy exceeding 90% and 92%. The Cellid_cell algorithm, which was evaluated by the gPRINT algorithm, also demonstrated high accuracy, exceeding 93%. The gPRINT algorithm is followed by the Cellid_cell algorithm, which is evaluated for accuracy (> 64%). These results indicate that the gPRINT algorithm is able to accurately map cell labels with a mixture of cell types and subtypes across time and datasets.

Additionally, the gPRINT algorithm was investigated for its potential to facilitate high-resolution mapping of cell subtype labels across diseases and datasets on the whole-body multi-tissue disease fibroblast atlas. The results demonstrate that the gPRINT algorithm achieves an average correct rate of 87.67% (Group), which is significantly higher than the next best algorithm, Cellid (Cell), at 67.72%. This indicates that the algorithm is more accurate than other algorithms in accomplishing cell subtype mapping across different disease subtypes. This will help to understand the composition of cells under different disease subtypes and their roles in the development of diseases. Furthermore, it will provide insights for targeted drug discovery and development. This will facilitate an understanding of the composition of cells under different disease subtypes and their roles in disease development and progression, as well as providing guidance for the development or exploration of targeted drugs.

Finally, we applied our methodology to tissue-specific fibrotic diseases. Initially, we obtained the human tendinopathy fibroblast subtype atlas through manual annotation. We then proceeded to complete the automatic annotation of human tendon fibroblast subtypes across health and disease. This process yielded insights into the differences in the osteochondrogenic tenocyte subtype and the myofibroblast subtype between tendinopathy patients and healthy individuals. The proportion of fibroblast subpopulations was higher in tendinopathy patients. This led us to hypothesize that the increase in the osteochondrogenic tenocyte subpopulation and Myofibroblast subpopulation might have contributed to the development of tendinopathy. To identify potential therapeutic targets, we analyzed the top 10 key differential genes from the drug database CMAP and key disease subtypes. The following genes were identified as key differential genes from the drug database CMAP and key disease subtypes: *MAP3K2*, *CALM1*, *MAP2K2*, *FKBP1A*, *MAPK1*, etc. A search was conducted for relevant drugs that can inhibit the expression of these genes, resulting in the identification of Axitinib, Sirolimus, Perphenazine, etc. This suggests that the automated annotation tool, gPRINT, can greatly improve the efficiency and accuracy of annotation of fibroblast subtype labels, thus advancing the research and therapeutic process of tendinopathy. Moreover, the subtype labeling of fibroblasts may vary among tendinopathy patients, suggesting that individualized and precise treatment is an important direction for tendinopathy treatment. The annotation of fibroblast subtype labels can provide a basis for the development of individualized therapeutic regimens, which can improve therapeutic efficacy and reduce therapeutic risks. Finally, annotation of fibroblast subtypes can also be used as one of the indicators for assessing the effectiveness of treatment. The detection and analysis of fibroblast subtype labels enables the assessment of treatment effectiveness and patient prognosis, thereby providing guidance for the development of subsequent treatment plans.

These findings illustrate that gPRINT not only provides a new method and idea for cell type annotation but also shows strong reliability and accuracy in cross-dataset type mapping with batch effect. In addition, the gPRINT algorithm was validated for cell type annotation in different disease states. These findings have important implications for understanding cell type differences in different health and disease states as well as the mechanisms of disease onset. Moreover, the methodology employed in this study can serve as a reference and provide insights for other cell type annotations and cell type mapping across datasets. In conclusion, the results of this study offer novel approaches and methodologies for analyzing and interpreting single-cell transcriptome data, which are crucial for elucidating cell type differences and the mechanisms of disease onset. Furthermore, they provide numerous avenues for future expansion and applications in cytological and biological research.

However, it is important to note that this study has limitations. Firstly, the choice of reference dataset can influence the accuracy and generalizability of the algorithm. Datasets with larger sample sizes and broader coverage are likely to yield more accurate annotation results. Secondly, the selection of algorithm and parameter settings can affect the stability and reliability of the results. Therefore, future studies should focus on further improving and refining the algorithms to enhance the accuracy and efficiency of cell type annotation and mapping across datasets. In the field of cell type annotation, further exploration of cell type differences across various tissues and organs, as well as changes in cell types associated with disease states, would be valuable. These studies could offer insights into the diversity and functions of cell types, which may, in turn, inform disease diagnosis and treatment. Additionally, with regard to cell type mapping across datasets, the algorithm should be validated on more datasets to improve its reliability and generalization. The algorithm could also be applied to single-cell data from other biological species to deepen our understanding of biodiversity. Methodologically, there is room to explore and improve techniques in data preprocessing, feature selection, and model construction to further enhance the accuracy and efficiency of cell type annotation and mapping. In conclusion, this study presents a novel method and concept for cell type annotation and mapping across datasets, with numerous potential avenues for expansion and future application. Furthermore, it is recommended that further exploration be undertaken to address the technical variations and discrepancies between different datasets, aiming to improve the reliability and generalizability of cell type mapping across diverse datasets.

## Methods

### Data preprocessing

All single-cell RNA sequencing (scRNA-seq) data were preprocessed using R (version 3.6.1). For single-cell data, deletions were made for genes with total expression less than two. When the dataset was used as a reference set, cell types with less than ten cells were deleted because the model obtained from training with too few cells would be overfitting. However, this was not required when the dataset was used as a test set. The genes in both the reference and test sets must be arranged according to the gene order of HG38 (by genomic termination site). The test set must include genes that are consistent with the reference set. If a gene is absent from the test set, a value of zero must be added to the data. The data must then be standardized and normalized using the NormalizeData() and ScaleData() functions in the Seurat package. This is done in preparation for running gPRINT methods.

In the event that the gPRINT_group algorithm was employed, the marker genes for each cell type were derived from a marker gene database (e.g., the PanglaoDB database [Franzén et al., 2019]) or calculated using the FindAllMarkers function of the Seurat package. These genes were then converted into a one-dimensional “feature print” based on the order of the HG38 reference genome and imported into the gPRINT_group model.

### Obtaining “gene print”

As is the case with each individual’s unique fingerprints and voiceprints, each cell also possesses its own distinctive “gene print.” The gPRINT algorithm maps the level of gene expression by reordering the genes expressed by each cell according to the termination sites of each gene in the human reference genome sequence, HG38, and then obtains the “gene print” to which the cell belongs. The gene expression level of each cell is mapped to obtain its own “gene print” pattern. The “gene print” is analogous to the amplitude envelope of a voiceprint (Kersta, 1962). Each gene may be regarded as a frame segment, with the expression value of the gene serving as the amplitude envelope value of the sound wave. Deep learning algorithms can then be employed for acoustic wave recognition to analyze the gene expression of a single cell. At present, deep learning has been extensively utilized in the fields of sound wave and audio recognition and classification, including sound classification, speech recognition, speech-to-text, intelligent assistant interaction, and communication. The most fundamental aspect of audio is the sound wave, which is a time series. The amplitude envelope extraction method is frequently employed for the extraction of features from the sound wave. The purpose of the amplitude envelope is to identify the maximum amplitude of each frame and then combine them into a single entity. The amplitude represents the volume (or loudness) of the signal. The signal is initially decomposed into its constituent windows, after which the maximum amplitude within each window is identified. The amplitude envelope is then formed by connecting the maximum amplitudes of each window along time. The majority of existing cellular annotation algorithms analyze or learn the cell-gene expression matrix directly. In contrast, our proposed algorithm, inspired by deep learning principles used in acoustic wave recognition, treats gene expression location information as the time domain of an acoustic wave, with each gene interval corresponding to a frame segment. This approach converts scRNA-seq data into waveform data for learning and annotation. In comparison to other algorithms, our method introduces a novel feature: the incorporation of gene position information.

It is well established that DNA is arranged in a sequential manner on each chromosome. Furthermore, histone proteins within the nucleosome, which serves as a spool of DNA entanglement, can influence gene transcription through post-translational modification at specific sites. Moreover, the genes within a nucleosome are clustered, and the genes within the same nucleosome may be co-regulated and exhibit similar expression patterns, resulting in the phenomenon of “clustered expression.” This phenomenon suggests that there may be a correlation between neighboring genes after the genes expressed are sorted according to the reference genome. Therefore, it is meaningful to add positional features for each cell. This additional dimension is incorporated into the algorithm to transform the gene expression pattern into a one-dimensional “gene print,” which introduces new features and aligns more closely with the form of gene expression. In principle, this should result in an improvement in the accuracy of cellular annotation.

### Construction of neural network methods for single-cell annotation

Based on the “gene print,” we use a six-layer 1-dimensional convolutional neural network (1D CNN), which consists of one input layer, five hidden layers and one output layer. The input layer has an equal number of nodes as the reference gene. The hidden layers contain two convolutional layers, one pooling layer, one flatten layer, and one fully connected layer. The purpose of the pooling layer is to retain the main features and reduce the computational load. The flatten layer is used to connect the convolutional layer to the dense fully connected layer. The main role of the fully connected layer is to nonlinearly vary the features extracted from the previous convolutional layer to extract the correlation between these features and map them to the output space. The number of genes in the reference dataset is used as the dimension of the model input, the number of convolution kernels in the convolutional layer is 16, the size of the convolution kernel is 3, and the softmax function is chosen for the dense layer.

Based on the “feature print,” we employ the back propagation (BP) neural network, which is a multi-layer feed-forward neural network. Its training process is mainly divided into two stages. The first stage is the forward propagation of the signal, and the second stage is the back propagation of the error. The learning rule employed is the most rapid descent method, which is used to continuously adjust the weights and thresholds of the network through back propagation to minimize the sum of error squares of the network.

### Parameter selection

We hope to use convolutional layers to simulate filters to achieve noise removal. Therefore, we chose convolutional neural networks (CNNs) as the basis of the model and tried to build convolutional networks with different numbers of layers. Following experimentation, it was found that when two convolutional layers were used, the model’s average accuracy and precision reached their highest value (93.01%) when five-fold cross-validation was performed on HCL single-cell data (20 tissues including Kidney, Epityphlon, Heart, Lung, Muscle, Ileum, Transverse_colon, JeJunum, Duodenum, etc.). This was achieved with the lowest variance (0.0016). These findings indicate that a CNN network comprising two convolutional layers exhibits optimal accuracy and stability in the cell type annotation task. Consequently, we have selected a CNN network with two convolutional layers as the foundation for the gPRINT algorithm (Fig. S1A; Tables S7 and S8).

We utilized convolutional models with 1–4 layers to perform 5-fold internal cross-validation on single-cell data from 20 tissues in the HCL, including the Ascending_colon, Bladder, Bone_marrow, Cervix, and so forth. Following the reordering of the genes, we find that the average accuracy of these models was found to be 2.88% higher than that observed with a random distribution. Furthermore, a significant difference was identified between the two groups (*P* = 0.028), which led to the selection of the reordered genes as the feature input for the model (Fig. S1B; Table S9).

### Evaluation metrics

In the context of machine learning for classification tasks, metrics such as precision, recall, accuracy, F1-score, and subject work curve are commonly employed for the evaluation of models. Among these, precision is defined as the proportion of true classes among the samples predicted to be positive classes, whereas recall, also known as the check-all rate, is the proportion of all positive classes that are predicted to be positive classes. Accuracy is a performance metric for evaluating classification problems. It is defined as the proportion of correctly classified samples to the total number of samples for a given set of data. F1-score, also known as the balanced F-score, is the reconciled average of the precision rate and the recall rate. It is a measure of the classification problem, with a maximum of 1 and a minimum of 0. The larger it is, the better the classification results are. The receiver operating characteristic is a graphical representation of the relationship between the false positive rate (FPR) and the true positive rate (TPR). The area under the curve (AUC) is a summary statistic that can be used to evaluate the classification performance of a model. The AUC ranges from 0 to 1, with a value closer to 1 indicating better classification performance.

For each cell type in each test dataset, the model was evaluated by calculating the following metrics: recall, precision, F1-score, and subject working curve. The formulas for these calculations are as follows:

The following formulas were used to calculate the precision, recall, accuracy, F1-score, and TPR for each cell type in each test dataset:

Precision = TP / (TP + FP)

Recall = TP / (TP + FN)

Accuracy = (TP + TN) / (TP + TN + FP + FN)

F1-score = 2TP / (2TP + FP + FN)

TPR (true positive rate) = TP / (TP + FN)

FPR (false positive rate) = FP / (FP + TN)

TP: True positive, indicates that the positive category is correctly identified as positive;

FP: False positive, indicates that the negative category is incorrectly identified as positive;

TN: True negative, indicates that the negative category is correctly identified as negative;

FN: False negative, indicates that the positive category is incorrectly identified as negative.

### Five-fold cross-validation

Cross-validation represents a crucial technique for evaluating models, whereby the dataset is partitioned into multiple subsets for training and validation purposes. This approach ensures the robustness and repeatability of the evaluation process. The fundamental premise is to mitigate model overfitting and selection bias by conducting repeated data splitting and model training evaluation, thereby obtaining more reliable performance estimates. This article employs the technique of five-fold cross-validation. The following section outlines the principal steps and methodologies employed to elucidate the cross-validation process.

The initial step in the cross-validation process is the division of the dataset. The dataset is divided into multiple subsets (referred to as “folds” or “blocks”). For example, 5-fold cross-validation divides the data into five subsets. The training and validation phases are as follows: Each iteration of the process should utilize k-1 subsets for training purposes, with the remaining subset employed for the purposes of validation. This process is repeated five times, with a different subset used for validation each time.

Step 1. The dataset is divided into five equal-sized subsets.

Step 2. Training and validation:

For each subset, the following steps are carried out:

Training: Four subsets are used to train the model.

Validation: The remaining subset is used to evaluate the model’s performance.

### Instructions for use

In the specific implementation, should the user wish to annotate the cell types or subtypes of pancreatic tissue cell types and fibrosis subtypes, which have been modeled in the original article, then it is only necessary to enter the gene expression matrix with “.csv” as the suffix and select the reference dataset name (e.g., “Pancreas”). Subsequently, the gPRINT_reference algorithm may be selected at the cell-to-cell level, or the gPRINT_group_reference algorithm may be selected at the group-to-group level. This will result in the direct retrieval of the annotation results for each cell, which will be presented in the form of an anno_result.csv file. Currently, our method includes pre-trained annotation models for pancreatic cell types. When annotating pancreatic data, users can choose whether to use the pre-trained model. In the future, we plan to expand the library to include pre-trained models for additional tissues, thereby broadening the scope of use.

For tissues without a pre-trained model, users must provide both a reference dataset and the test dataset to perform label mapping between the two. First, users should input the gene expression matrices and annotation-related cell information (with csv suffix) for each dataset. In the test set, annotation-related cell information refers to cell names, while in the reference dataset, annotation-related cell information corresponds to cell types or subtypes labels. Thereafter, the gPRINT algorithm should be selected at the cell to cell level, or the gPRINT_group algorithm at the group to group level. This will result in the direct retrieval of the annotation results of each cell, which are stored in the anno_result.csv file.

## Supplementary Material

pwaf001_suppl_Supplementary_Figures_S1-S10

pwaf001_suppl_Supplementary_Table_S1

pwaf001_suppl_Supplementary_Table_S2

pwaf001_suppl_Supplementary_Table_S3

pwaf001_suppl_Supplementary_Table_S4

pwaf001_suppl_Supplementary_Table_S5

pwaf001_suppl_Supplementary_Table_S6

pwaf001_suppl_Supplementary_Table_S7

pwaf001_suppl_Supplementary_Table_S8

pwaf001_suppl_Supplementary_Table_S9

## Data Availability

Our laboratory has previously accumulated multiple validated single-cell transcriptome datasets that can be used for model validation and application testing. For example, the raw sequence data for single-cell datasets related to tendinopathy have been deposited in the Genome Sequence Archive (Genomics, Proteomics & Bioinformatics 2021) in National Genomics Data Center (Nucleic Acids Res 2024), China National Center for Bioinformation/Beijing Institute of Genomics, Chinese Academy of Sciences (GSA-Human: HRA001687), and are publicly accessible. Additionally, we can access single-cell transcriptome data from various tissues and organs through the Gene Expression Omnibus (GEO) database. Specifically, datasets from Wang, Xin, and Muraro, Baron datasets for pancreatic scRNA-seq, Xi dataset for muscle and tendon-related data, Li dataset for human colorectal tumor data, Camp dataset for human brain tissue-related single-cell data, human non-small cell lung cancer (NSCLC) disease subgroup maps, and their associated cell type annotations were downloaded from GEO (GSE83139, GSE81608, GSE85241, GSE84133, GSE147457, GSE81861, GSE75140, and GSE153935). The dataset from Segerstolpe dataset was obtained from ArrayExpress (EBI) (E-MTAB-5061). Notably, the Wang and Xin datasets used the SMARTer method for single-cell RNA sequencing, the Muraro dataset utilized CEL-Seq2, and the Segerstolpe dataset was generated using Smart-Seq2 sequencing. For human whole-body multi-tissue fibrosis atlas, it can be accessed through fibroXplorer webpage. The human pancreatic cancer single-cell data is available in the EGA database (EGAD00001005365). Many single-cell datasets from various tissues are sourced from the Human Cell Landscape (HCL) database.

## Code availability

The source code and results are available in GitHub. (github.com/Yanyanruojin/gPRINT).
