# Supplementary material for: Gene print-based cell subtypes annotation of human disease across heterogeneous datasets with gPRINT
Source: Protein Cell. 2025 Mar 14;16(8):685–704. doi: 10.1093/procel/pwaf001 (PMC12342163; doi:10.1093/procel/pwaf001)
Supplement: pwaf001_suppl_Supplementary_Figures_S1-S10 [file pwaf001_suppl_supplementary_figures_s1-s10.pdf]

Supplementary material

Figure S1. The basis for selecting parameters of the gPRINT algorithm.

Figure S2. Schematic and diagram of the algorithm.

Figure S3. Test results for the internal test set.

Figure S4. A line chart illustrating the evaluation metrics for the annotation accuracy of different algorithms applied to single-cell data from tissues such as Blood, Brain, Liver, and Pancreas in the HCL dataset.

Figure S5. tSNE plot presentation of the annotation results of different algorithms for the Wk12_14 and Wk17_18 datasets.

Figure S6. Application of annotations across time on the adult skeletal muscle atlas.

Figure S7. Presentation of the atlas of systemic multi-tissue fibrosis disease.

Figure S8. Fibroblast subtype mapping analysis associated with each subtype of NSCLC disease.

Figure S9. Results of cross-dataset cell subtype annotation for breast cancer.

Figure S10. Schematic diagram of CMap's search for tendinopathy-related drugs.

Supplementary Table S1. Cell type table of pancreas datasets

Supplementary Table S2. Statistical table of indicators of each annotation algorithm in the cross-dataset evaluation of various organizations.

Supplementary Table S3. The top 150 upregulated differential genes between healthy samples and tendon disease samples.

Supplementary Table S4. The top 150 downregulated differentially expressed genes between healthy samples and tendon disease samples.

Supplementary Table S5. The obtained normalized connectivity score (NCS) values related to CMap algorithms.

Supplementary Table S6. The 100 small molecules shown in gray in Figure S9.

Supplementary Table S7. Evaluation indicators of five-fold cross-validation of different convolutional layer models on various organizations

Supplementary Table S8. The mean and variance of evaluation indicators of different convolutional layers in Table S7.

Supplementary Table S9. Statistical table comparing the difference in evaluation indicators of annotation results after five-fold cross validation when reordered genes and randomly distributed genes are used as model inputs.

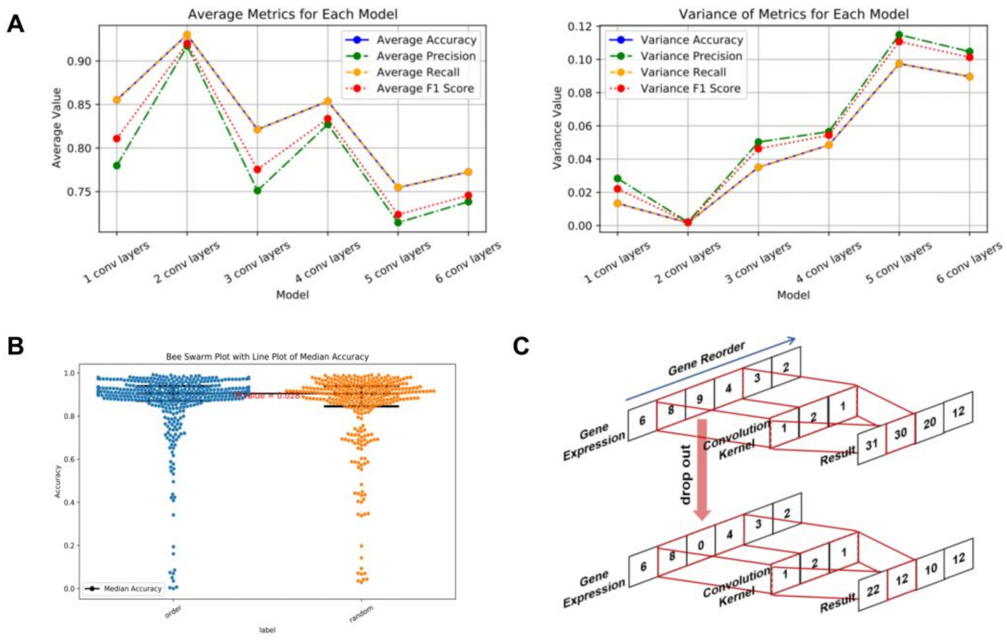

Figure S1. The basis for selecting parameters of the gPRINT algorithm. A) Line graph of the average and variance of the accuracy of the models constructed by different convolutional layers. B) Comparison of the accuracy of the gene reordering and random gene permutation groups, P<0.05. C) Schematic diagram of the results of convolution kernel calculation for gene expression sequences without dropout and with dropout events after gene reordering.

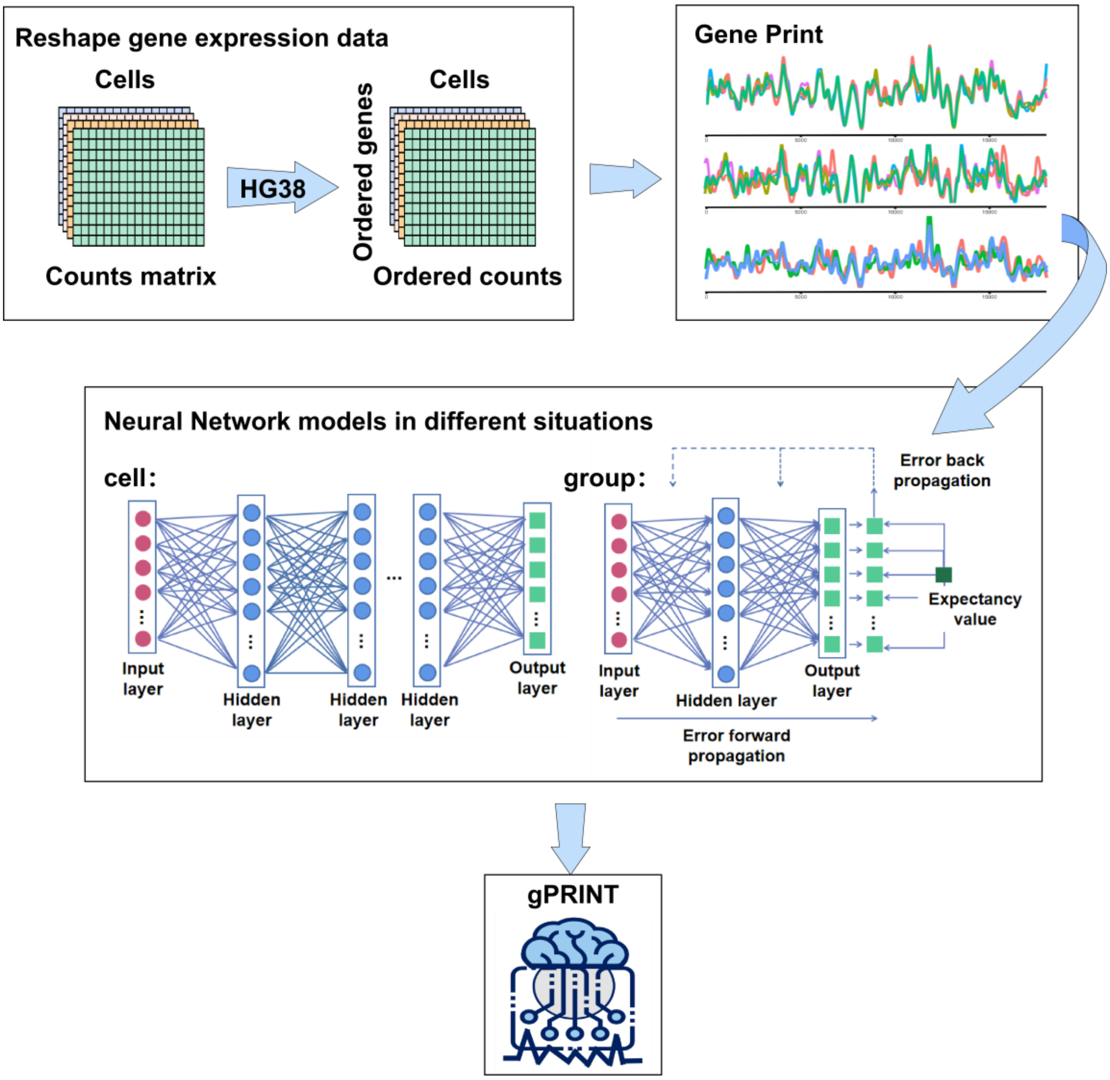

Figure S2. Schematic and diagram of the algorithm.

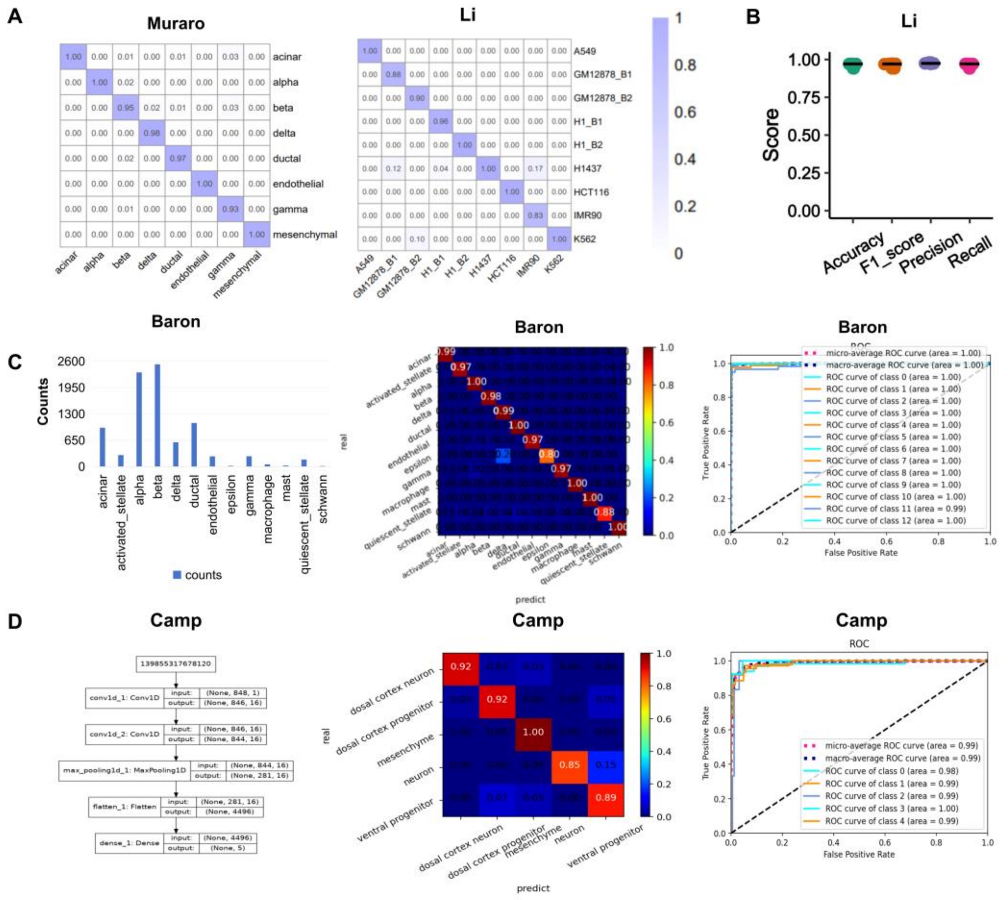

Figure S3. Test results for the internal test set. (A) Muraro dataset and Li dataset's Confusion matrix presentation. (B) Swarm diagram of Li dataset. (C) Bar chart depicting cell type information from the Baron dataset, along with the confusion matrix and ROC curve of the annotation results. (D) Model composition during training on the Camp dataset, along with the confusion matrix and ROC curve of the annotation results.

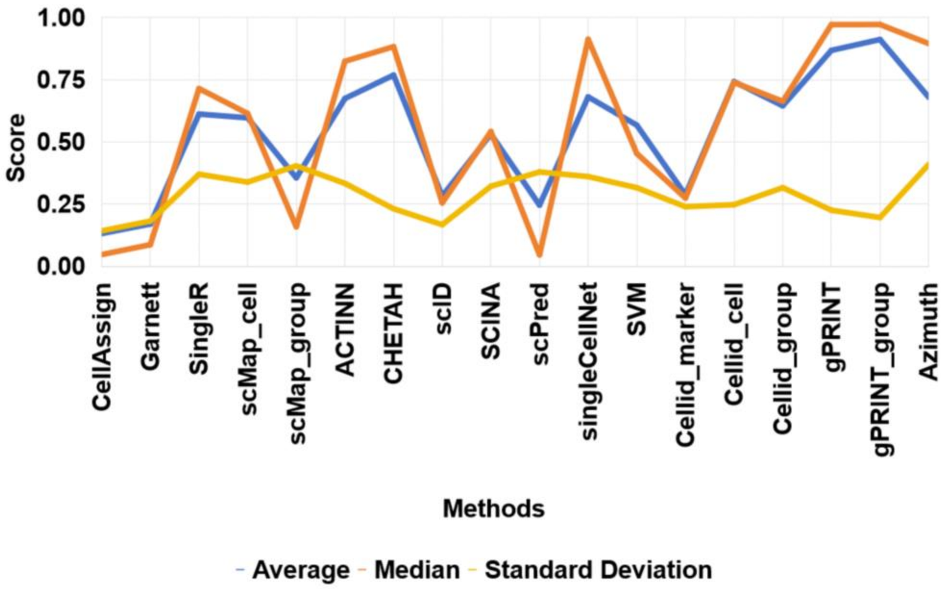

Figure S4. A line chart illustrating the evaluation metrics for the annotation accuracy of different algorithms applied to single-cell data from tissues such as Blood, Brain, Liver, and Pancreas in the HCL dataset.

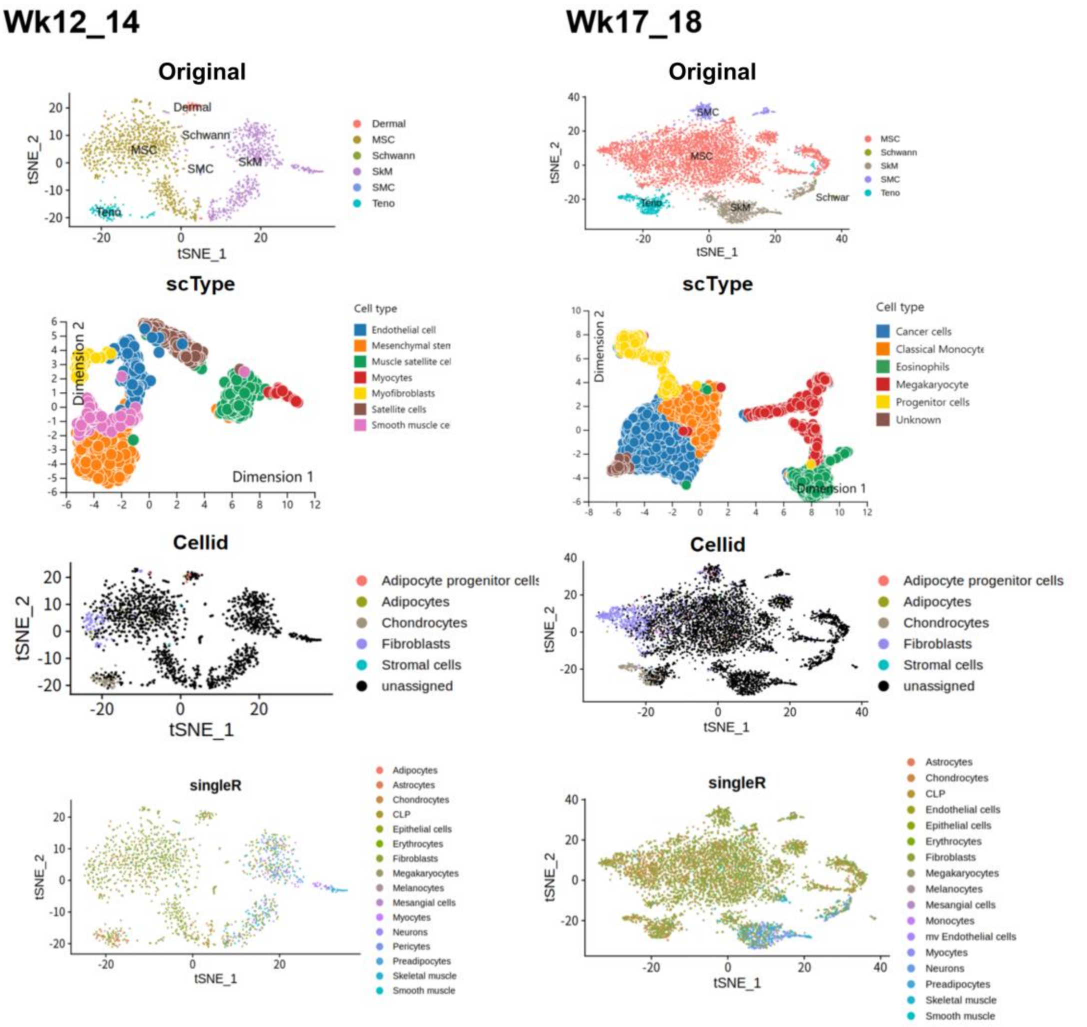

Figure S5. tSNE plot presentation of the annotation results of different algorithms for the Wk12_14 and Wk17_18 datasets.

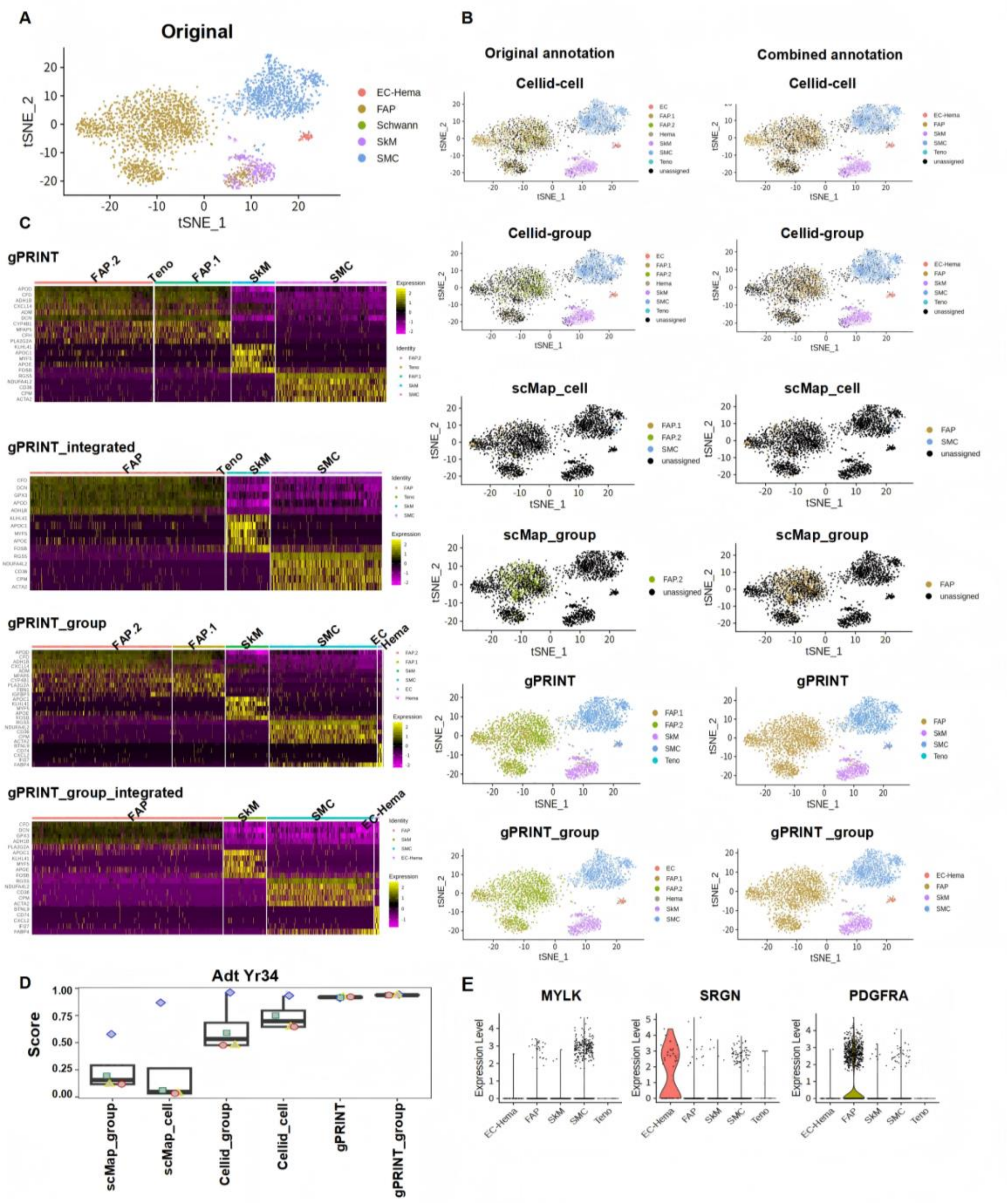

Figure S6. Application of annotations across time on the adult skeletal muscle atlas. (A) t-SNE plot of cell distribution of the Adult Yr34_42 dataset in the original article on the Adult Skeletal Muscle Atlas, where the color of the cells represents the cell type. (B) t-SNE plot demonstrating the annotation results of different annotation methods on Adult Yr34-42, where the color of the cells represents the cell type. The original annotation results are shown on the left, and the integrated annotation results are shown on the right (C) Heatmap display of the top 5 differential expressions in the cell types obtained from all gPRINT correlation predictions (D) Histograms of evaluation metrics for each annotation algorithm on the samples of the Adult Yr34-42 dataset.

Boxplots summarize method's F1 scores, and show the median (center line), interquartile range (hinges) and 1.5 times the interquartile range (whiskers). (E) Violin plots indicating the expression levels of marker genes for all different cell types on the cell subtypes obtained by different annotation methods.

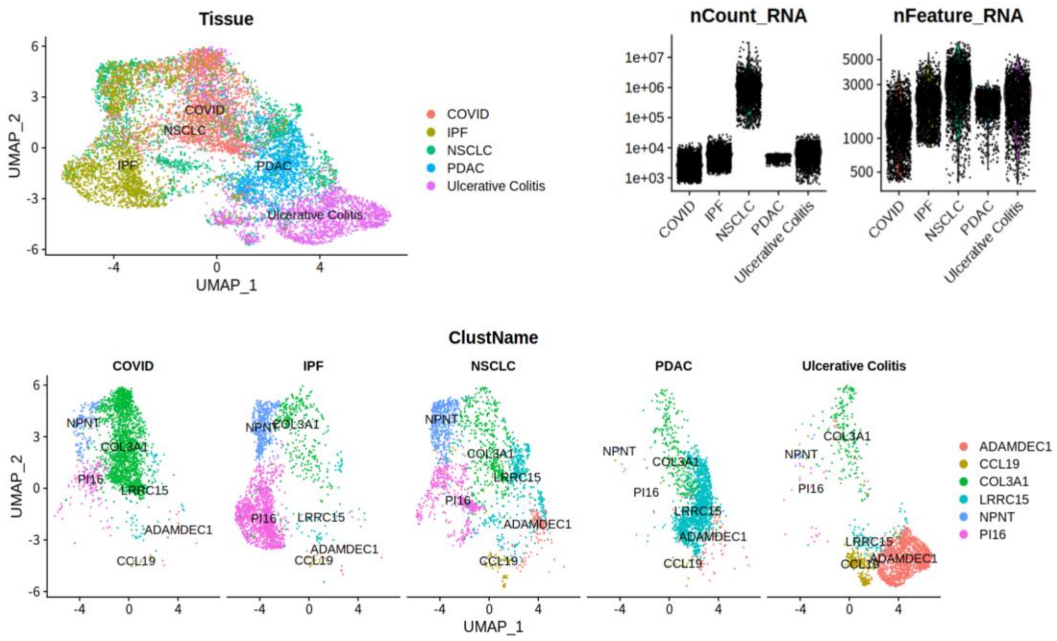

Figure S7. Presentation of the atlas of systemic multi-tissue fibrosis disease.

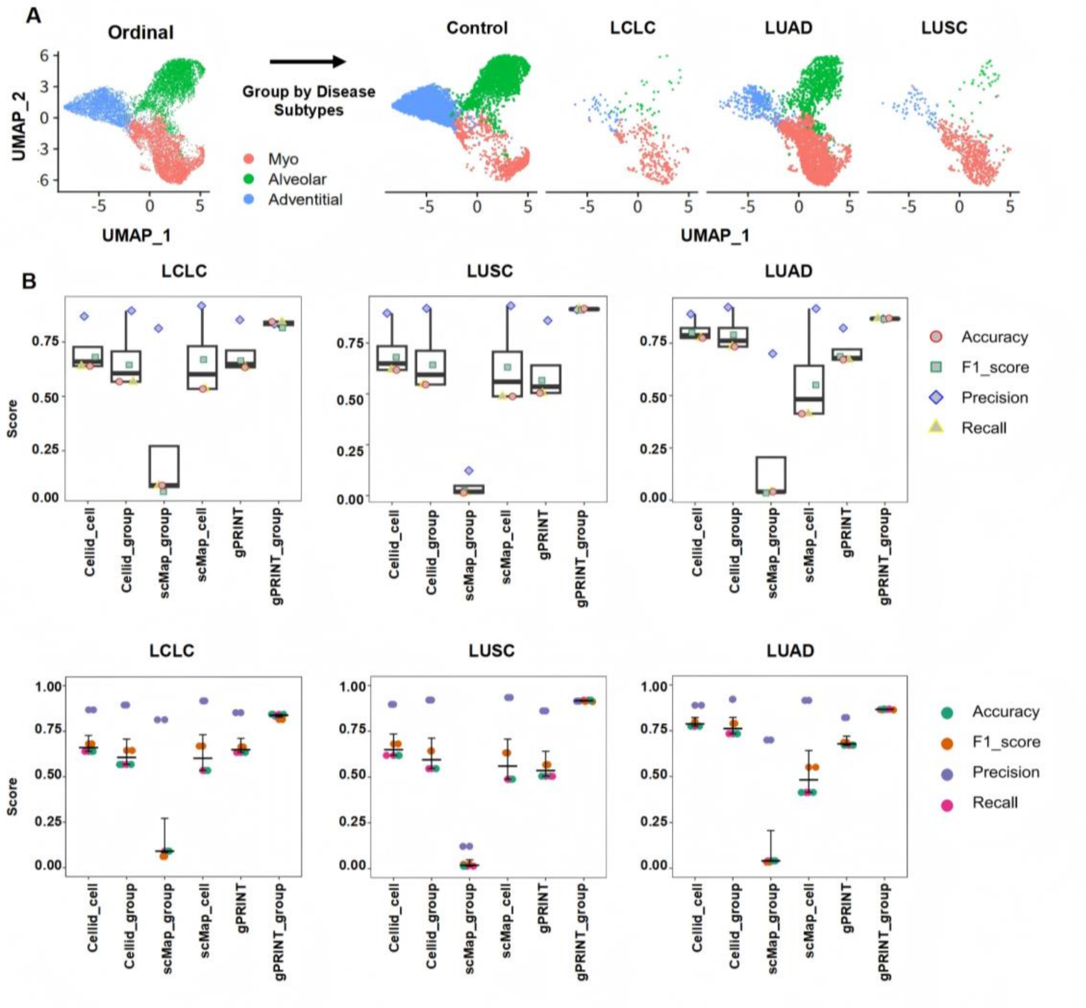

Figure S8. Fibroblast subtype mapping analysis associated with each subtype of NSCLC disease.

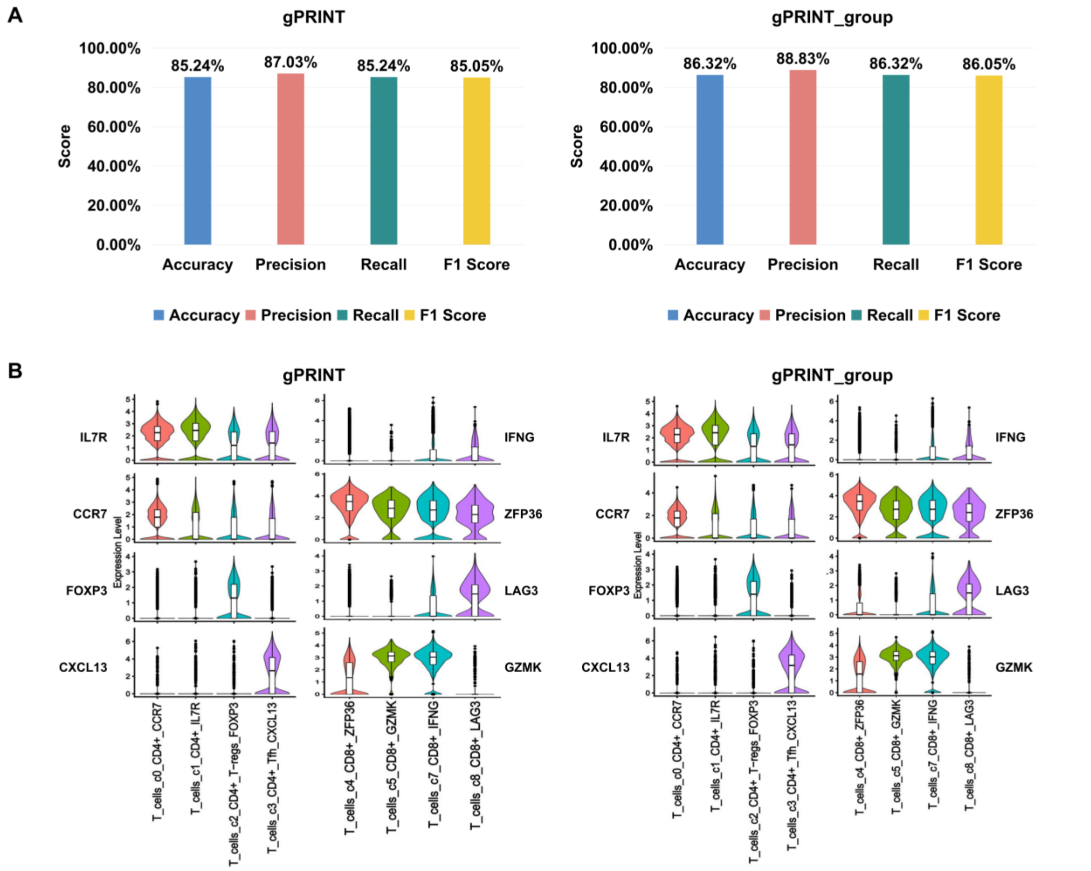

Figure S9. Results of cross-dataset cell subtype annotation for breast cancer. A) The bar graph shows the annotation results of gPRINT and gPRINT_group algorithms on CD8, CD4 and other subtypes of T cells in the NC dataset based on the NG dataset. B) The violin plot shows the expression of cell subtypes annotated by gPRINT and gPRINT_group algorithms on their respective marker genes.

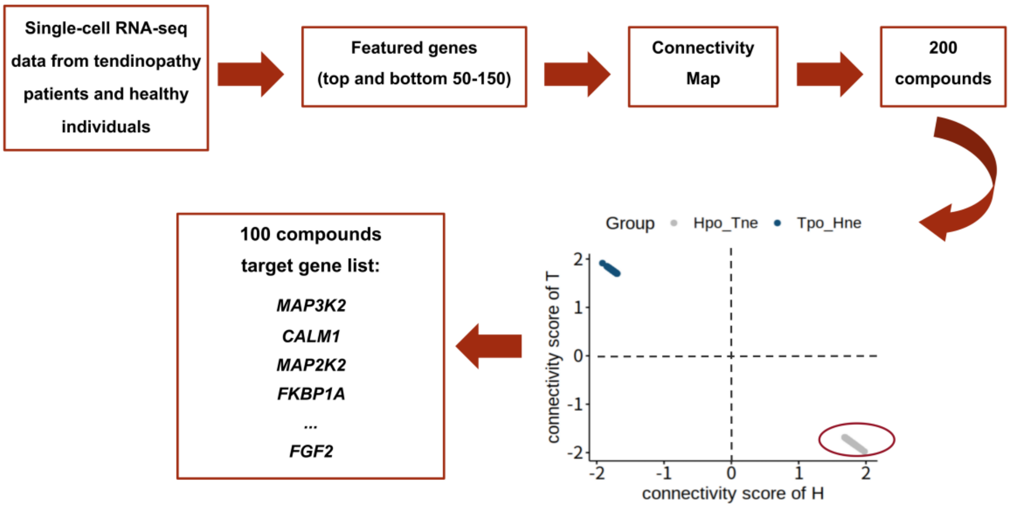

Figure S10. Schematic diagram of CMap's search for tendinopathy-related drugs.
